# Racial and ethnic disparities in mortality among World Trade Center Health Registry enrollees with post‐9/11 cancer

**DOI:** 10.1002/cam4.70071

**Published:** 2024-08-27

**Authors:** Rebecca D. Kehm, Jiehui Li, James E. Cone

**Affiliations:** ^1^ New York City Department of Health and Mental Hygiene World Trade Center Health Registry Long Island City New York USA; ^2^ Department of Epidemiology, Mailman School of Public Health Columbia University New York New York USA

**Keywords:** 9/11‐disaster, disparity, mortality, post‐9/11 cancer, race and ethnicity, socioeconomic status

## Abstract

**Introduction:**

There are well‐documented racial and ethnic disparities in mortality after cancer in the general population, but less is known about whether disparities also exist in disaster‐exposed populations.

**Methods:**

We conducted a longitudinal cohort study of 4341 enrollees in the World Trade Center Health Registry (WTCHR) with a first‐ever primary invasive cancer diagnosis after 9/11/2001 and followed through 2020. We examined associations of race and ethnicity with all‐cause mortality risk and cause‐specific mortality risk using multivariable Cox proportional hazards regression models and Fine and Gray's proportional sub‐distribution hazards models, respectively. Models were adjusted for baseline characteristics and tumor characteristics. We also examined models further adjusted for socioeconomic status (SES), and we used inverse odds weighting to formally test for mediation by SES.

**Results:**

Compared to non‐Hispanic White enrollees with cancer, non‐Hispanic Blacks had higher risks for all‐cause mortality (adjusted hazard ratio (aHR) = 1.20, 95% CI = 1.02–1.41) and non‐cancer mortality (aHR = 1.48, 95% CI = 1.09–2.01) in the full model. In the model without SES, Hispanic enrollees with cancer had higher risks for all‐cause mortality (aHR = 1.32, 95% CI = 1.09–1.60) and cancer mortality (aHR = 1.31, 95% CI = 1.05–1.64) compared to non‐Hispanic Whites; these associations became not statistically significant in the full model. In the inverse odds weighting analysis, SES explained 24% and 29% of the disparity in all‐cause mortality risk observed in non‐Hispanic Blacks and Hispanics, respectively, compared to non‐Hispanic Whites.

**Conclusion:**

This study found that there are racial and ethnic disparities in mortality after cancer in the WTCHR. Additional studies are needed to further explore the factors mediating these disparities.

## Graphical abstract

This study among 9/11‐exposed individuals with post‐9/11 cancer found that, compared with non‐Hispanic White enrollees, non‐Hispanic Black enrollees had statistically significantly higher risk for non‐cancer mortality, and Hispanic enrollees had higher risk for cancer‐related mortality. The mortality disparity between Hispanic and non‐Hispanic White enrollees was mediated by socioeconomic status.

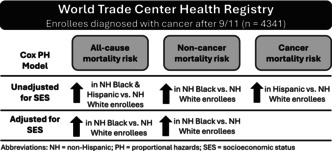

## 1 INTRODUCTION

There are well documented racial and ethnic disparities in cancer mortality in the United States.^1^, ^2^, ^3^ A 2022 report by the American Cancer Society found that the overall cancer death rate was 19% higher in non‐Hispanic Black (Black) males compared to non‐Hispanic White (White) males and 12% higher in Black females compared to White females.^4^ These disparities are thought to be due, at least in part, to upstream social determinants of health that lead to worse health outcomes in communities of color and other marginalized groups.^4^, ^5^, ^6^ This is supported by the fact that the 5‐year survival rate for all cancers combined was found to be 10% lower in US adults living in counties with ≥20% of the population below the federal poverty line compared to those living in more affluent counties (i.e., <10% below the poverty line).^1^ A study using data from the California Cancer Registry found that neighborhood‐level socioeconomic status (SES) and marital status accounted for 5%–18% and 6%–21%, respectively, of the survival gap between racial and ethnic groups for the most common types of adult cancers, including prostate, breast, lung, and colorectal.^7^ Another study using data from the Surveillance Epidemiology and End Results (SEER) database found that neighborhood‐level SES accounted for 28%–73% of the survival gap between Black and Hispanic children with cancer compared to White children with cancer.^8^ These studies support that SES may be an important mediating factor contributing to racial and ethnic disparities in mortality after cancer, at least in the general population.

Less is known about whether racial and ethnic disparities in mortality after cancer exist in disaster exposed populations in which there is a shared experience of trauma and other harmful exposures. This includes both man‐made disasters such as industrial accidents and terrorist attacks, as well as disasters due to natural hazards such as hurricanes and earthquakes.^9^ For example, few studies have considered whether there are racial and ethnic disparities in mortality after cancer among individuals who were exposed the World Trade Center (WTC) terrorist attacks on September 11, 2001 (9/11), which has been associated with increased risk for certain cancers.^10^, ^11^, ^12^, ^13^ A recent mortality study of a combined cohort of WTC rescue and recovery (rescue/recovery) workers showed that Black enrollees had significantly higher risks for all‐cause mortality, heart disease mortality, and smoking‐related mortality, but not for cancer‐related mortality, compared to White enrollees, though the primary exposure of interest was level of WTC exposure.^14^ Yet, this study was limited in scope in that it did not include those who were also exposed to the 9/11 but were not involved in rescue/recovery efforts, nor did it examine mortality risk specifically among enrollees diagnosed with cancer after September 11, 2001.

A better understanding of whether there are racial and ethnic differences in survival after cancer in the WTC exposed population, as well as the extent to which SES contributes to survival differences, may be important for developing interventions that can mitigate disparities. A study of WTC Health Registry (WTCHR) enrollees with cancer found that 9/11‐related post‐traumatic stress disorder (PTSD) was associated with increased risk of all‐cause mortality in enrollees diagnosed with cancer after, but not before, September 11, 2001.^15^ It is thus possible that racial and ethnic disparities in mortality exist specifically among enrollees diagnosed with cancer after September 11, 2001, given that other studies have identified racial and ethnic differences in risk for 9/11‐related PTSD. For example, a recent study found that the prevalence of 9/11‐related PTSD was higher in Black and Hispanic enrollees versus White enrollees (17% and 22%, respectively, vs. 7%) in the WTCHR.^16^ Another study of WTC responders found that non‐traditional responders (i.e., utility workers, asbestos cleaners, construction workers, administrators, volunteers, transportation workers) and traditional responders (i.e., police) of Hispanic ethnicity had higher risk for 9/11‐related PTSD compared to their White and Black counterparts, which was explained by differences in income and education level.^17^ We thus hypothesized that there are racial and ethnic disparities in mortality among WTCHR enrollees diagnosed with cancer after September 11, 2001, which may be explained by differences in SES. To test this hypothesis, we examined associations of race and ethnicity with risk for all‐cause mortality and cause‐specific mortality among WTCHR enrollees with cancer after September 11, 2001. We examined associations overall and stratified by WTC rescue/recovery worker status, and we examined potential mediation by SES.

## 2 METHODS

### 2.1 Study population

We used data from the WTCHR, a cohort of individuals exposed to the WTC terrorist attacks on September 11, 2001 (details published elsewhere^18^, ^19^). The cohort includes rescue/recovery workers and volunteers who worked at the WTC site, debris‐loading sites, on barges, or at the Staten Island landfill, and non‐rescue/recovery workers who lived, worked, went to school or were passersby in lower Manhattan, defined as south of Canal Street, on September 11, 2001. Over 71,000 exposed individuals (42.9% rescue/recovery workers, 57.1% non‐rescue/recovery workers) enrolled in the WTCHR and completed a baseline survey between 2003 and 2004.^18^ For this analysis, we included enrollees with a first‐ever primary invasive (or in situ bladder) cancer diagnosis between September 11, 2001 and December 31, 2015 reported by 10 state cancer registries selected for linkage (New York, New Jersey, California, Connecticut, North Carolina, Massachusetts, Ohio, Pennsylvania, Texas, and Washington; *n* = 5467). At least 93% of the WTCHR enrollees lived in the catchment areas of these selected registries at some point during follow‐up. All 10 states started their cancer registries in 1996 or earlier and had complete cancer incidence records available through 2015 at the time of linkage. Cancer sites were defined using SEER site recode *International Classification for Oncology, Third Edition* (ICD‐O‐3) groups.^20^ We excluded individuals with a first primary cancer diagnosis before age 20 years (*n* = 12), a baseline survey completed by a proxy (*n* = 104), a missing cancer diagnosis date (*n* = 24) and missing data on smoking status and/or PTSD check list (PCL) (*n* = 86). This results in a final analytic sample size of 4341 enrollees.

This study was approved by the Institutional Review Board of the NYC Department of Health and Mental Hygiene (DOHMH). Each cancer registry record linkage was also approved by the respective IRB of 10 state departments of health and the Rutgers University of New Jersey. A Federal Certificate of Confidentiality was obtained, and oral informed consent was obtained for participants at enrollment.

### 2.2 Ascertainment of deaths

Deaths were identified through December 31, 2020 via data linkage to the NYC DOHMH Vital Records and the National Death Index. Underlying cause of death was classified using International Classification of Disease codes, 10th revision (ICD‐10). Deaths for which cancer was listed as the underlying cause of death were counted as events in analyses of cancer‐specific mortality; all other deaths were counted as events in analyses of non‐cancer mortality.

### 2.3 Self‐reported measures from questionnaires

Race and ethnicity were self‐reported on the baseline survey and categorized as White, Black, Hispanic, and other (includes non‐Hispanic Asian or Pacific Islander, Native American, and other enrollee responses). White enrollees with cancer served as the reference group in analyses. Consistent with our previous study of mortality after cancer in the WTCHR,^15^ we created a 3‐level SES variable derived from baseline data on household income and educational attainment: low (defined as <$50,000 household income and a college degree or less), medium (defined as <$50,000 and more than a college degree; $50,000–$74,999 and a college degree or less; or $74,999–$149,999 and a high school degree or less), and high (defined as $75,000–$149,999 and a college degree or more; or ≥$150,000 and any education). These categories were selected based on the distribution of crude death rates across the household income and education categories. Additional baseline covariates, selected a priori following literature review, included gender (female, male), smoking status (current, former, never), enrollee group, pre‐9/11 physical health conditions, and 9/11‐related PTSD. Enrollees were grouped as rescue/recovery workers or non‐rescue/recovery workers. Non‐rescue/recovery workers included residents, area workers, passersby, local school staff and students. We derived a count (0, 1, 2, ≥3) of pre‐9/11 physical health conditions, including asthma, hypertension, heart condition (angina, myocardial infarction, heart disease, or other heart condition), stroke, emphysema, and diabetes. PTSD was evaluated on the baseline survey using the PTSD Checklist‐Specific (PCL‐S), which consists of 17 Likert items (scored 1 = not at all to 5 = extremely) corresponding to three symptom clusters (re‐experiencing, avoidance, hyperarousal) from the Diagnostic and Statistical Manual of Mental Disorders (DSM‐IV).^21^, ^22^ We defined PTSD as a PCL‐S score of ≥50 to increase specificity and minimize false‐positives.^23^

### 2.4 Tumor and prognostic characteristics from cancer registries

Variables related to the first cancer diagnosis were obtained from cancer registries and included age at diagnosis, diagnosis year, cancer site, stage at diagnosis, grade, and subsequent cancer diagnoses (any/none). To account for cancer types that have better prognoses and are more amenable to treatment than others, we categorized tumor treatment amenability of first cancers by their 5‐year relative survival rates based on SEER18 data^24^ and previously defined cut points.^25^

### 2.5 Statistical analyses

We used Cox proportional hazards regression to calculate hazard ratios (HRs) and 95% confidence intervals (CIs) for the associations of race and ethnicity with all‐cause mortality risk among WTCHR enrollees diagnosed with cancer after September 11, 2001. We used Fine and Gray's proportional subdistribution hazard models^26^ to calculate HRs and 95% CIs for the associations of race and ethnicity with cancer‐specific mortality risk and non‐cancer (deaths from causes other than cancer) mortality risk. Person‐time from first cancer diagnosis to death or censoring (December 31, 2020) was used as the underlying time scale. Follow‐up time was left truncated at the date of the baseline survey in those diagnosed with cancer between September 11, 2001 and the date of study enrollment to avoid potential survival bias. We examined associations in three separate models: (1) unadjusted for covariates; (2) adjusted for gender, smoking status at baseline, PCL score at baseline, pre‐9/11 physical health conditions, tumor stage at diagnosis, tumor grade at diagnosis, tumor treatment amenability (5‐year relative survival rates), and subsequent cancer diagnoses (i.e., adjusted model without SES); and (3) further adjusted for SES (i.e., fully adjusted model with SES). We assessed the proportional hazards assumption with Schoenfeld residuals methods,^27^ and identified no significant violations of the assumption except for age at diagnosis. Therefore, we stratified all models by age at diagnosis using the STRATA statement in SAS to allow baseline hazard functions to vary across age groups.^27^ We repeated the above analysis stratified by enrollee group, given that we identified differences in mortality risks between rescue/recovery workers and non‐rescue/recovery workers in our WTCHR study.^15^

For all‐cause mortality risk, we further explored potential mediation by SES using the inverse odds weighting (IOW) method.^28^, ^29^ IOW is a semiparametric, weight‐based approach that can be used for any functional form and is valid even in the presence of exposure‐mediator interactions.^28^, ^29^ We used the IOW method to estimate the total effect, natural direct effect, and natural indirect effect of race and ethnicity on all‐cause mortality. We obtained standard errors for these estimates through bootstrapping (500 replications). We used these estimates to calculate the percent change from the total effect to the natural direct effect (β_total_−β_direct_)/β_total_. Details on the IOW method, including the assumptions of this approach, are provided elsewhere.^28^, ^29^ We were not able to use the IOW method to explore mediation for cause‐specific mortality due to the smaller number of events in these analyses. Statistical analyses were performed using SAS, v9.4 (SAS Institute Inc., Cary, NC). All statistical tests were 2‐sided and *p* < 0.05 were considered statistically significant.

### 2.6 Sensitivity analyses

We conducted a sensitivity analysis excluding breast and prostate cancer cases to examine if findings were primarily driven by these common cancers. We also conducted a sensitivity analysis excluding deaths occurring after March 2020, given that there may have been a temporary spike in non‐cancer deaths related to COVID‐19 during this period. Finally, we conducted a sensitivity analysis in the rescue/recovery workers subgroup in which we stratified by self‐reported use (ever/never) of WTC Medical Monitoring and Treatment Program (MMTP) services based on self‐reported data from four subsequent follow‐up surveys (2006 to 2020). This analysis was done to explore whether differential utilization of MMTP services contributes to racial and ethnic disparities in mortality after cancer among rescue/recovery workers.^14^, ^30^

## 3 RESULTS

### 3.1 Study sample characteristics

We observed 1335 deaths (1011 cancer‐related deaths and 324 non‐cancer deaths) over 39,425.1 person‐years of follow‐up. There were differences in baseline characteristics across the racial and ethnic groups (Table 1). This included differences in gender, smoking status, enrollee group, SES, pre‐9/11 physical health conditions, and post‐9/11 PTSD. Tumor‐related characteristics also differed by race and ethnicity, including age at diagnosis, year of diagnosis, 5‐year relative survival rate of cancer type, tumor stage at diagnosis, and tumor grade at diagnosis (Table 2).

**TABLE 1:** Baseline demographic characteristics by race and ethnicity among enrollees with cancer after 9/11 in the World Trade Center Health Registry, *N* = 4341.

Characteristic at baseline	Race and ethnicity				*p*‐Value^b^
	Non‐Hispanic White	Non‐Hispanic Black	Hispanic	Other^a^	
	(*n* = 3050)	(*n* = 639)	(*n* = 376)	(*n* = 276)	
	*n* (%)	*n* (%)	*n* (%)	*n* (%)	
Gender					
Female	989 (32.4)	315 (49.3)	170 (45.2)	135 (48.9)	<0.0001
Male	2061 (67.6)	324 (50.7)	206 (54.8)	141 (51.1)	
Smoking status					
Never	1333 (43.7)	356 (55.7)	200 (53.2)	193 (69.9)	<0.0001
Former	1245 (40.8)	172 (26.9)	106 (28.2)	52 (18.8)	
Current	472 (15.5)	111 (17.4)	70 (18.6)	31 (11.2)	
Enrollee group					
Rescue and recovery workers	1378 (45.2)	203 (31.8)	148 (39.7)	39 (14.1)	<0.0001
Non‐rescue and recovery workers^c^	1672 (54.8)	436 (68.2)	228 (60.6)	237 (85.9)	
Socioeconomic status (SES)^d^					
Low	449 (14.7)	279 (43.7)	190 (50.3)	136 (49.3)	<0.0001
Moderate	748 (24.5)	148 (23.2)	90 (23.9)	42 (15.2)	
High	1479 (48.5)	140 (21.9)	66 (17.6)	61 (22.1)	
Unknown	374 (12.3)	72 (11.3)	30 (8.0)	37 (13.4)	
Pre‐9/11 physical health conditions (excluding cancer)					
None	2025 (66.4)	373 (58.4)	245 (65.2)	168 (60.9)	<0.01
1 condition	781 (25.6)	197 (30.8)	89 (23.7)	77 (27.9)	
2+ conditions	244 (8.0)	69 (10.8)	42 (11.2)	31 (11.2)	
9/11‐related PCL score					
<50	2804 (91.9)	515 (80.6)	265 (70.5)	244 (88.4)	<0.0001
≥50	246 (8.1)	124 (19.4)	111 (29.5)	32 (11.6)	

Abbreviation: PCL, posttraumatic stress disorder check list.

^a^ Other includes non‐Hispanic Asian or Pacific Islander, Native American, and other participant responses.

^b^ *p*‐Value is reported from a 2‐sided Pearson Chi‐squared test.

^c^ Include residents, area workers, passersby, and school staff and students not involved in WTC rescue and recovery effort.

^d^ A 3‐level socioeconomic status (SES) variable was derived from baseline data on household income and educational attainment: low (defined as <$50,000 household income and a college degree or less), medium (defined as <$50,000 and more than a college degree; $50,000–$74,999 and a college degree or less; or $74,999–$149,999 and a high school degree or less), and high (defined as $75,000–$149,999 and a college degree or more; or ≥$150,000 and any education). These categories were selected based on the distribution of crude death rates across the household income and education categories.

**TABLE 2:** Vital status and clinical characteristics by race and ethnicity among enrollees with cancer after 9/11 in the World Trade Center Health Registry, *N* = 4341.

Vital status, and characteristic at diagnosis	Race and ethnicity				*p*‐Value^b^
	Non‐Hispanic White	Non‐Hispanic Black	Hispanic	Other^a^	
	(*n* = 3050)	(*n* = 639)	(*n* = 376)	(*n* = 276)	
	*n* (%)	*n* (%)	*n* (%)	*n* (%)	
Vital status					
Alive	2176 (71.3)	422 (66.0)	242 (64.4)	166 (60.1)	<0.0001
Deceased, cancer‐related cause of death	668 (21.9)	155 (24.3)	103 (27.4)	85 (30.8)	
Deceased, non‐cancer cause of death	206 (6.8)	62 (9.7)	31 (8.2)	25 (9.1)	
Sum of person‐years of follow‐up	28,450.2	5716.8	3089.0	2169.1	
Age at diagnosis, years					
<40	205 (6.7)	24 (3.8)	22 (5.9)	13 (4.7)	<0.0001
40–49	522 (17.1)	119 (18.6)	57 (15.2)	44 (15.9)	
50–59	1047 (34.3)	244 (38.2)	132 (35.1)	70 (25.4)	
60–69	906 (29.7)	193 (30.2)	118 (31.4)	62 (22.5)	
≥70	370 (12.1)	59 (9.2)	47 (12.5)	87 (31.5)	
Subsequent cancer diagnoses					
Yes	259 (8.5)	41 (6.4)	24 (6.4)	19 (6.9)	0.17
No	2791 (91.5)	598 (93.6)	352 (93.6)	257 (93.1)	
Tumor treatment amenability^c^					
Low	517 (17.0)	102 (16.0)	73 (19.4)	77 (27.9)	<0.0001
Medium	550 (18.0)	110 (17.2)	72 (19.2)	70 (25.4)	
High	1983 (65.0)	427 (66.8)	231 (61.4)	129 (46.7)	
Top five cancer sites^d^					
Prostate	697 (22.9)	196 (30.7)	74 (19.7)	34 (12.3)	NA
Breast	364 (11.9)	126 (19.7)	62 (16.5)	38 (13.8)	
Lung and bronchus	211 (6.9)	42 (6.6)		35 (12.7)	
Skin melanomas	188 (6.2)				
Thyroid	160 (5.3)	24 (3.8)	21 (5.6)	17 (6.2)	
Corpus uteri		22 (3.4)	22 (5.9)		
Liver			23 (6.1)	13 (4.7)	
Stage at diagnosis					
Localized	1756 (57.6)	359 (56.2)	199 (52.9)	127 (46.0)	<0.0001
Regional	602 (19.7)	131 (20.5)	87 (23.1)	57 (20.7)	
Distant	533 (17.5)	118 (18.5)	66 (17.6)	56 (20.3)	
Unknown	159 (5.2)	31 (4.9)	24 (6.4)	36 (13.0)	
Grade					
Well differentiated (I)	297 (9.7)	59 (9.2)	37 (9.8)	27 (9.8)	<0.01
Moderately differentiated (II)	827 (27.1)	201 (31.5)	107 (28.5)	69 (25.0)	
Poorly or undifferentiated (III‐IV)	771 (25.3)	202 (31.6)	87 (23.1)	69 (25.0)	
Other (V–VIII)	233 (7.6)	42 (6.7)	30 (8.0)	19 (6.9)	
Unknown/not applicable	922 (30.2)	135 (21.1)	115 (30.6)	92 (33.3)	

Abbreviation: NA, Not applicable.

^a^ Other includes non‐Hispanic Asian or Pacific Islander, Native American, and other participant responses.

^b^ *p*‐Value is reported from a two‐sided Pearson Chi‐square test.

^c^ Tumor treatment amenability estimated based on the 5‐year relative survival rate for each cancer type calculated using Surveillance Epidemiology and End Results 18 data: Low, <40% 5‐year survival rate, Medium, 40%–69% 5‐year survival rate, and High, ≥70% 5‐year survival rate.

^d^ Based on the five most common cancer sites diagnosed in each racial and ethnic group; the types of cancers and their ordering differs for each racial and ethnic group.

### 3.2 Associations of race and ethnicity with all‐cause mortality risk

Black and Hispanic enrollees with cancer had a higher risk for all‐cause mortality compared to White enrollees with cancer in the model without SES (Table 3; adjusted hazard ratio (aHR) = 1.33, 95% CI = 1.14–1.55 and aHR = 1.32, 95% CI = 1.09–1.60, respectively). In the full model, only the Black‐White comparison was statistically significant (aHR = 1.20, 95% CI = 1.02–1.41). All‐cause mortality risk did not differ between other racial and ethnic groups compared to White enrollees with cancer. The findings of sensitivity analysis excluding breast and prostate cancers (Table S1), as well as the sensitivity analysis excluding deaths after March 2020 (Table S2), were consistent with the main findings.

**TABLE 3:** Associations of race and ethnicity with all‐cause and cause‐specific mortality risks among enrollees with cancer after 9/11 in the World Trade Center Health Registry, *N* = 4341.

Outcome	Unadjusted model^a^	Adjusted model without SES^b^	Fully adjusted model with SES^c^
Race and ethnicity	HR (95% CI)	HR (95% CI)	HR (95% CI)
All‐cause mortality			
Non‐Hispanic White	ref.	ref.	ref.
Non‐Hispanic Black	1.29 (1.11, 1.50)	1.33 (1.14, 1.55)	1.20 (1.02–1.41)
Hispanic	1.32 (1.11, 1.59)	1.32 (1.09, 1.60)	1.18 (0.97–1.43)
Other^d^	1.18 (0.96, 1.44)	1.03 (0.83, 1.27)	0.93 (0.74–1.15)
Cancer mortality			
Non‐Hispanic White	ref.	ref.	ref.
Non‐Hispanic Black	1.16 (0.97, 1.38)	1.15 (0.95, 1.41)	1.07 (0.87–1.31)
Hispanic	1.30 (1.06, 1.60)	1.31 (1.05, 1.64)	1.20 (0.96–1.52)
Other^d^	1.25 (0.99, 1.58)	1.08 (0.83, 1.40)	0.99 (0.76–1.29)
Non‐cancer mortality			
Non‐Hispanic White	ref.	ref.	ref.
Non‐Hispanic Black	1.62 (1.22, 2.15)	1.66 (1.23, 2.22)	1.48 (1.09–2.01)
Hispanic	1.23 (0.84, 1.80)	1.12 (0.75, 1.67)	1.00 (0.66–1.51)
Other^d^	0.84 (0.54, 1.30)	0.82 (0.52, 1.30)	0.73 (0.45–1.17)

Abbreviations: CI, confidence interval; HR, hazard ratio; ref., referent; SES, socioeconomic status.

^a^ Model is stratified by age group at diagnosis, but unadjusted for covariates.

^b^ Model is stratified by age group at diagnosis and adjusted for gender, enrollee group, smoking status, PCL score, stage at diagnosis, grade at diagnosis, amenability, second cancer, and pre‐911 physical health conditions.

^c^ Model is stratified by age group at diagnosis and adjusted for gender, enrollee group, smoking status, PCL score, stage at diagnosis, grade at diagnosis, amenability, second cancer, pre‐911 physical health conditions, and socioeconomic status.

^d^ Other includes non‐Hispanic Asian or Pacific Islander, Native American, and other participant responses.

**FIGURE 1:**
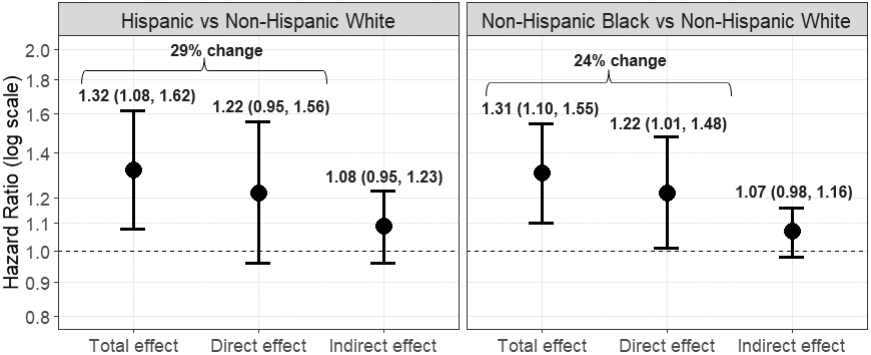
Inverse odds weighting analysis of mediation by socioeconomic status of the associations between race and ethnicity and all‐cause mortality risk among enrollees with cancer after 9/11 in the World Trade Center Health Registry. Hazard ratios and 95% confidence intervals are estimated from multivariable Cox proportional hazards regression models that are adjusted for enrollee group, smoking status, PCL score, stage at diagnosis, grade at diagnosis, amenability, second cancer, pre‐911 physical health conditions, and age at diagnosis (continuous). The model estimating the direct effect is also weighted to account for the association between race and ethnicity and socioeconomic status; weights were estimated in separate models for the non‐Hispanic Black versus non‐Hispanic White association and the Hispanic versus non‐Hispanic White association. The indirect effects were calculated by subtracting the direct effect from the total effect, and standard errors were bootstrapped with 500 replications. The precent change was calculated as follows: (βtotal–βdirect)/βtotal.

Using the IOW method, we found that SES explained 29% and 24% of the disparity in all‐cause mortality risk observed in Black and Hispanic enrollees, respectively, compared to White enrollees (Figure 1). The direct and indirect effect estimates were similar for the Black‐White and Hispanic‐White comparisons, although only the direct effect for the Black‐White comparison was statistically significant (aHR = 1.22, 95% CI = 1.01–1.48).

### 3.3 Associations of race and ethnicity with cause‐specific mortality risk

Black enrollees with cancer had a higher non‐cancer mortality risk compared to White enrollees in the full model (aHR = 1.48, 95% CI = 1.09–2.01). Cancer mortality risk did not differ between Black and White enrollees with cancer. Hispanic enrollees with cancer had a higher cancer mortality risk compared to White enrollees in the model without SES (aHR = 1.31, 95% CI = 1.05–1.64), but not in the full model (aHR = 1.20, 95% CI = 0.96–1.52). Non‐cancer mortality risk did not differ between Hispanic and White enrollees with cancer. Cause‐specific mortality risks did not differ between other racial and ethnic groups and White enrollees.

### 3.4 Associations of race and ethnicity with mortality risk by enrollee group

Among non‐rescue/recovery workers with cancer, all‐cause mortality and non‐cancer mortality risks were higher in Black enrollees compared to White enrollees in the full model (Table 4; aHR = 1.29, 95% CI = 1.06–1.56 and aHR = 1.68, 95% CI = 1.15–2.45, respectively). All‐cause mortality risk was higher in Hispanic versus White non‐rescue/recovery workers with cancer in the model without SES (aHR = 1.34, 95% CI = 1.06–1.69), but not in the full model (aHR = 1.23, 95% CI = 0.98–1.57). Overall, mortality risks did not differ by race and ethnicity among rescue/recovery workers with cancer. However, among rescue/recovery workers who reported never using MMTP services, Black versus White enrollees had a higher cancer mortality risk in the full model (Table S3; aHR = 1.62, 95% CI = 1.00–2.61). By contrast, among rescue/recovery workers who used MMTP services, Black versus White enrollees had a lower cancer mortality risk in the full model (aHR = 0.30, 95% CI = 0.10–0.86). Among rescue/recovery workers who used MMTP services, other racial and ethnic groups had a higher cancer mortality risk compared to their White counterparts in the full model (aHR = 4.80, 95% CI = 1.73–13.28).

**TABLE 4:** Associations of race and ethnicity with all‐cause and cause‐specific mortality risks by enrollee group among enrollees with cancer after 9/11 in the World Trade Center Health Registry, *N* = 4341.

	Non‐rescue and recovery workers (*n* = 2573)			Rescue and recovery workers (*n* = 1768)		
Outcome	Unadjusted model^a^	Adjusted model without SES^b^	Fully adjusted model with SES^c^	Unadjusted model^a^	Adjusted model without SES^b^	Fully adjusted model with SES^c^
Race and ethnicity	HR (95% CI)	HR (95% CI)	HR (95% CI)	HR (95% CI)	HR (95% CI)	HR (95% CI)
All‐cause mortality						
Non‐Hispanic White	ref.	ref.	ref.	ref.	ref.	ref.
Non‐Hispanic Black	1.46 (1.22–1.72)	1.39 (1.15–1.67)	1.29 (1.06–1.56)	0.97 (0.73–1.29)	1.17 (0.87–1.58)	1.02 (0.75–1.38)
Hispanic	1.38 (1.11–1.72)	1.34 (1.06–1.69)	1.23 (0.98–1.57)	1.18 (0.85–1.63)	1.31 (0.94–1.85)	1.13 (0.80–1.60)
Other^d^	1.22 (0.98–1.52)	1.03 (0.82–1.30)	0.96 (0.75–1.22)	0.85 (0.42–1.72)	1.11 (0.54–2.28)	1.01 (0.49–2.09)
Cancer mortality						
Non‐Hispanic White	ref.	ref.	ref.	ref.	ref.	ref.
Non‐Hispanic Black	1.27 (1.02–1.57)	1.16 (0.91–1.47)	1.10 (0.86–1.41)	0.91 (0.66–1.27)	1.22 (0.83–1.78)	1.09 (0.74–1.60)
Hispanic	1.36 (1.06–1.74)	1.32 (1.00–1.74)	1.24 (0.93–1.66)	1.13 (0.78–1.65)	1.34 (0.93–1.93)	1.18 (0.81–1.73)
Other^d^	1.27 (0.99–1.63)	1.11 (0.83–1.47)	1.05 (0.78–1.40)	0.93 (0.44–1.97)	0.97 (0.49–1.90)	0.89 (0.42–1.87)
Non‐cancer mortality						
Non‐Hispanic White	ref.	ref.	ref.	ref.	ref.	ref.
Non‐Hispanic Black	1.86 (1.33–2.61)	1.88 (1.32–2.68)	1.68 (1.15–2.45)	1.19 (0.69–2.03)	1.14 (0.63–2.04)	1.07 (0.59–1.93)
Hispanic	1.28 (0.81–2.02)	1.10 (0.67–1.79)	0.97 (0.58–1.62)	1.21 (0.60–2.41)	1.24 (0.61–2.52)	1.15 (0.56–2.33)
Other^d^	0.92 (0.57–1.47)	0.89 (0.55–1.45)	0.79 (0.47–1.33)	0.52 (0.07–3.85)	0.59 (0.07–4.92)	0.56 (0.07–4.72)

Abbreviations: CI, confidence interval; HR, hazard ratio; ref., referent; SES, socioeconomic status.

^a^ Model is stratified by age group at diagnosis, but unadjusted for covariates.

^b^ Model is stratified by age group at diagnosis and adjusted for gender, smoking status, PCL score, stage at diagnosis, grade at diagnosis, amenability, second cancer, and pre‐911 physical health conditions.

^c^ Model is stratified by age group at diagnosis and adjusted for gender, smoking status, PCL score, stage at diagnosis, grade at diagnosis, amenability, second cancer, pre‐911 physical health conditions, and socioeconomic status.

^d^ Other includes non‐Hispanic Asian or Pacific Islander, Native American, and other participant responses.

## 4 DISCUSSION

This is the first study to examine racial and ethnic disparities in mortality after cancer in WTCHR rescue/recovery workers and non‐rescue/recovery workers. Overall, before stratifying by rescue/recovery worker status, we found that Black and Hispanic enrollees with cancer had 33% and 32%, respectively, higher risk for all‐cause mortality compared to White enrollees with cancer before accounting for SES. After adjustment for SES, these associations were attenuated, and the disparity in all‐cause mortality between Hispanic and White enrollees was no longer statistically significant. All‐cause mortality risk did not differ between other racial/ethnic groups and White enrollees with cancer, regardless of their rescue/recovery worker status. Results from the IOW analysis further supported that differences in SES may explain some, but not all, of the observed racial and ethnic disparities in all‐cause mortality risk among WTCHR enrollees with cancer. Specifically, SES accounted for 24% and 29% of the respective Black‐White and Hispanic‐White disparities in all‐cause mortality risk. These findings suggest that, besides SES, there may be other factors (e.g., physical inactivity, diet, and cancer screening utilization)^2^ contributing to racial and ethnic disparities in mortality risk.

When we examined cause‐specific mortality, we found that risk for non‐cancer mortality was higher in Black versus White enrollees, while risk for cancer mortality was higher in Hispanic versus White enrollees. For cause‐specific mortality, we were not able to formally test for mediation by SES using the IOW method due to small case counts. However, we found that the disparity in cancer mortality between Hispanic and White enrollees became not statistically significant after adjustment for SES, suggesting that social factors may play a major role in this observed association. Further studies should explore whether socially patterned factors such as delayed access to care, treatment adherence, and health care quality, are contributing to cancer mortality risk in Hispanic enrollees. Additional studies are also needed to better understand what is leading to the increased risk for non‐cancer mortality in Black versus White enrollees in the WTCHR. For example, multiple studies in the general population have found that Black cancer patients experience higher rates of cardiotoxicity from chemotherapy than White cancer patients, which increases risk for cardiovascular disease‐related mortality.^31^ Because SES was not a significant mediator of the observed disparities in mortality between Black and White enrollees in this analysis, future WTCHR studies should explore other non‐SES related factors (e.g., exposure to racial discrimination, health behaviors) that may contribute to non‐cancer mortality risk among Black enrollees with cancer.

When we examined associations stratified by enrollee group, we observed racial and ethnic differences in mortality risks only among non‐rescue/recovery workers with cancer. No overall racial and ethnic differences in mortality risks were observed among rescue/recovery workers with cancer. It is possible that racial and ethnic disparities are mitigated among rescue/recovery workers due to more homogeneity in terms of employment status, as well as underlying health status due to stringent pre‐employment health screenings and required fitness levels for many job categories (e.g., firefighters, police officers). Further, rescue/recovery workers had access to health services that were not always available to non‐rescue/recovery workers,^32^ which may also reduce racial and ethnic health disparities. For example, firefighters working for the FDNY were enrolled in a WTC health program that began on September 11, 2001 as an extension of a pre‐existing occupational health program,^33^ and general responders were enrolled in a separate health program (i.e., Medical Monitoring and Treatment Program, MMTP) established in July 2002 for non‐FDNY WTC workers and volunteers established in 2002.^34^, ^35^ In the sensitivity analysis of rescue/recovery workers by MMTP service use, we found that cancer mortality risk was higher in Black versus White rescue/recovery workers among MMTP service non‐users, but not among MMTP service users. We also found that, among rescue/recovery workers who used MMTP services, cancer mortality risk was nearly 5‐fold higher among other racial and ethnic groups compared to Whites. While these findings should be interpreted with caution given the wide confidence interval around estimates, they provide interesting preliminary data suggesting that access and utilization of MMTP services may contribute to racial and ethnic disparities in the WTCHR. Further studies should explore whether MMTP services are being equally utilized across racial and ethnic groups in the WTCHR, and whether differences in MMTP utilization contribute to racial and ethnic disparities in mortality after cancer.

This study has several strengths, the primary one being that it is the first study to examine racial and ethnic disparities in mortality after cancer in the WTCHR, which is important for understanding health equity in disaster‐exposed populations. There are also limitations to this study. We used self‐reported data from baseline survey to evaluate SES and other covariates. This may have led to misclassification bias, especially given that SES and other covariates (e.g., PTSD) may change over time and thus not accurately reflect measures from around the time of cancer diagnosis. Further, SES is a multidimensional construct that might not be fully captured by our composite measure of education and household income. We also did not capture neighborhood‐level SES in this analysis, which has been linked to mortality after cancer in previous studies.^7^, ^8^ Future studies should thus explore the role of area‐level factors in racial and ethnic disparities in the WTCHR. Other social determinants of health, such as exposure to discrimination, should also be explored in future WTCHR studies. Another study limitation is that we did not use the IOW method to evaluate mediation by SES for cause‐specific mortality due to small sample size within subgroups. Small case counts all prevented us from further stratifying the cause of death beyond cancer versus non‐cancer deaths. Future WTCHR studies with additional years of follow‐up data should thus continue to explore racial and ethnic disparities in mortality after cancer.

### 4.1 Conclusions

This study supports that Black and Hispanic enrollees have higher all‐cause mortality risks after cancer than White enrollees, although these disparities were driven by different underlying causes of death (i.e., Black enrollees had higher non‐cancer mortality risk than Whites, while Hispanic enrollees had higher cancer mortality risk than Whites). Future WTCHR studies should continue to monitor racial and ethnic differences in mortality after cancer, and further explore the underlying factors mediating these disparities.

## AUTHOR CONTRIBUTIONS

**Rebecca D. Kehm:** Conceptualization (equal); methodology (lead); writing – original draft (lead); writing – review and editing (equal). **Jiehui Li:** Conceptualization (equal); data curation (lead); formal analysis (lead); funding acquisition (equal); writing – review and editing (equal). **James E. Cone:** Conceptualization (equal); funding acquisition (equal); writing – review and editing (equal).

## CONFLICT OF INTEREST STATEMENT

The authors declare no conflicts of interest.

## DISCLAIMERS

Its contents are solely the responsibility of the authors. There are additional disclaimers specified by individual State Cancer Registry: “The ideas and opinions expressed herein are those of the author(s) and do not necessarily reflect the opinions of the State of California, Department of Public Health, the National Cancer Institute, and the Centers for Disease Control and Prevention or their Contractors and Subcontractors”. “The Connecticut Department of Public Health does not endorse or assume any responsibility for any analyses, interpretations or conclusions based on the data. The authors assume full responsibility for all such analyses, interpretations and conclusions”. “Use of these data does not imply that Ohio Department of Health (ODH) or the Centers for Disease Control and Prevention (CDC) agrees or disagrees with the analyses, interpretations or conclusions in this publication”, “The Pennsylvania Department of Health specifically disclaims responsibility for any analyses, interpretations or conclusions”.

## Supporting information


Table S1.

## Data Availability

World Trade Center Health Registry data may be made available following review of applications to the Registry from external researchers. Cancer data from 10 state cancer registries may be requested from those entities separately. The data are not publicly available due to privacy or ethical restrictions.
