# Supplementary material for: Racial and ethnic disparities in mortality among World Trade Center Health Registry enrollees with post‐9/11 cancer
Source: Cancer Med. 2024 Aug 27;13(16):e70071. doi: 10.1002/cam4.70071 (PMC11348902; doi:10.1002/cam4.70071)
Supplement: Supplementary file 1 — Table S1. [file CAM4-13-e70071-s001.docx]

| **Supplementary eTable 1.** Associations of race and ethnicity with all-cause and cause-specific mortality risks, excluding prostate and breast cancer cases, among enrollees with cancer after 9/11 in the World Trade Center Health Registry, N=2,750 | | | |
| --- | --- | --- | --- |
| **Outcome** | Unadjusted  model^a^ | Adjusted model without SES^b^ | Fully adjusted model with SES^c^ |
| Race and ethnicity | HR (95% CI) | HR (95% CI) | HR (95% CI) |
| **All-cause mortality** |  |  |  |
| Non-Hispanic White | ref. | ref. | ref. |
| Non-Hispanic Black | 1.49 (1.25-1.79) | 1.34 (1.11-1.61) | 1.23 (1.01-1.49) |
| Hispanic | 1.28 (1.04-1.57) | 1.26 (1.02-1.57) | 1.15 (0.92-1.43) |
| Other^d^ | 0.21 (0.97-1.50) | 1.10 (0.88-1.38) | 1.00 (0.80-1.27) |
| **Cancer mortality** |  |  |  |
| Non-Hispanic White | ref. | ref. | ref. |
| Non-Hispanic Black | 1.37 (1.11-1.68) | 1.18 (0.94-1.49) | 1.11 (0.87-1.41) |
| Hispanic | 1.25 (0.99-1.56) | 1.30 (1.01-1.66) | 1.21 (0.94-1.55) |
| Other^d^ | 1.21 (0.95-1.54) | 1.07 (0.82-1.39) | 1.00 (0.76-1.30) |
| **Non-cancer mortality** |  |  |  |
| Non-Hispanic White | ref. | ref. | ref. |
| Non-Hispanic Black | 1.52 (1.03-2.25) | 1.50 (0.99-2.26) | 1.40 (0.91-2.14) |
| Hispanic | 1.11 (0.68-1.78) | 1.00 (0.60-1.67) | 0.93 (0.55-1.57) |
| Other^d^ | 0.93 (0.56-1.55) | 0.93 (0.55-1.56) | 0.85 (0.49-1.47) |
| Abbreviations: CI = confidence interval; HR = hazard ratio; ref. = referent; SES = socioeconomic status | | | |
| ^a^Model is stratified by age group at diagnosis, but unadjusted for covariates. | | | |
| ^b^Model is stratified by age group at diagnosis and adjusted for gender, enrollee group, smoking status, PCL score, stage at diagnosis, grade at diagnosis, amenability, second cancer, and pre-911 physical health conditions. | | | |
| ^c^Model is stratified by age group at diagnosis and adjusted for gender, enrollee group, smoking status, PCL score, stage at diagnosis, grade at diagnosis, amenability, second cancer, pre-911 physical health conditions, and socioeconomic status. | | | |
| ^d^Other includes non-Hispanic Asian or Pacific Islander, Native American, and other participant responses. | | | |

| **Supplementary eTable 2.** Associations of race and ethnicity with all-cause and cause-specific mortality risks, excluding deaths after March 2020, among enrollees with cancer after 9/11 in the World Trade Center Health Registry, N=4,277 | | | |
| --- | --- | --- | --- |
| **Outcome** | Unadjusted  model^a^ | Adjusted model without SES^b^ | Fully adjusted model with SES^c^ |
| Race and ethnicity | HR (95% CI) | HR (95% CI) | HR (95% CI) |
| **All-cause mortality** |  |  |  |
| Non-Hispanic White | ref. | ref. | ref. |
| Non-Hispanic Black | 1.29 (1.10-1.50) | 1.36 (1.16-1.59) | 1.22 (1.03-1.44) |
| Hispanic | 1.32 (1.09-1.59) | 1.33 (1.10-1.62) | 1.18 (0.96-1.44) |
| Other^d^ | 1.21 (0.99-1.49) | 1.06 (0.85-1.31) | 0.94 (0.76-1.18) |
| **Cancer mortality** |  |  |  |
| Non-Hispanic White | ref. | ref. | ref. |
| Non-Hispanic Black | 1.18 (0.99-1.41) | 1.18 (0.96-1.44) | 1.09 (0.89-1.55) |
| Hispanic | 1.33 (1.08-1.64) | 1.34 (1.06-1.69) | 1.23 (0.97-1.34) |
| Other^d^ | 1.28 (1.02-1.61) | 1.10 (0.85-1.42) | 1.01 (0.77-1.31) |
| **Non-cancer mortality** |  |  |  |
| Non-Hispanic White | ref. | ref. | ref. |
| Non-Hispanic Black | 1.65 (1.23-2.23) | 1.67 (1.22-2.29) | 1.48 (1.07-2.06) |
| Hispanic | 1.20 (0.79-1.82) | 1.11 (0.73-1.70) | 0.97 (0.63-1.52) |
| Other^d^ | 0.74 (0.45-1.21) | 0.80 (0.49-1.32) | 0.70 (0.42-1.18) |
| Abbreviations: CI = confidence interval; HR = hazard ratio; ref. = referent; SES = socioeconomic status | | | |
| ^a^Model is stratified by age group at diagnosis, but unadjusted for covariates. | | | |
| ^b^Model is stratified by age group at diagnosis and adjusted for gender, enrollee group, smoking status, PCL score, stage at diagnosis, grade at diagnosis, amenability, second cancer, and pre-911 physical health conditions. | | | |
| ^c^Model is stratified by age group at diagnosis and adjusted for gender, enrollee group, smoking status, PCL score, stage at diagnosis, grade at diagnosis, amenability, second cancer, pre-911 physical health conditions, and socioeconomic status. | | | |
| ^d^Other includes non-Hispanic Asian or Pacific Islander, Native American, and other participant responses. | | | |

| **Supplementary eTable 3.** Associations of race and ethnicity with all-cause and cause-specific mortality risks by self-reported use of Medical Monitoring and Treatment Program services among rescue and recovery workers with cancer after 9/11 in the World Trade Center Health Registry, N=1,500^a^ | | | | | | | |
| --- | --- | --- | --- | --- | --- | --- | --- |
|  | Ever Used MMTP Services (n=738) | | |  | Never Used MMTP Services (n=762) | | |
| **Outcome** | Unadjusted model^b^ | Adjusted model without SES^c^ | Fully adjusted model with SES^d^ |  | Unadjusted model^b^ | Adjusted model without SES^c^ | Fully adjusted model with SES^d^ |
| Race and ethnicity | HR (95% CI) | HR (95% CI) | HR (95% CI) |  | HR (95% CI) | HR (95% CI) | HR (95% CI) |
| **All-cause mortality** |  |  |  |  |  |  |  |
| Non-Hispanic White | ref. | ref. | ref. |  | ref. | ref. | ref. |
| Non-Hispanic Black | 0.60 (0.31-1.16) | 0.69 (0.35-1.36) | 0.66 (0.32-1.33) |  | 1.04 (0.70-1.56) | 1.42 (0.93-2.18) | 1.24 (0.80-1.93) |
| Hispanic | 0.86 (0.40-1.87) | 1.32 (0.57-3.04) | 1.22 (0.52-2.89) |  | 1.28 (0.78-2.08) | 1.06 (0.63-1.79) | 0.95 (0.56-1.62) |
| Other^e^ | 1.13 (0.28-4.62) | 2.74 (0.62-12.06) | 2.53 (0.57-11.25) |  | 1.00 (0.41-2.43) | 0.94 (0.36-2.46) | 0.87 (0.33-2.33) |
| **Cancer mortality** | |  |  |  |  |  |  |
| Non-Hispanic White | ref. | ref. | ref. |  | ref. | ref. | ref. |
| Non-Hispanic Black | 0.26 (0.09-0.79) | 0.31 (0.11-0.88) | 0.30 (0.10-0.86) |  | 1.09 (0.69-1.72) | 1.74 (1.09-2.78) | 1.62 (1.00-2.61) |
| Hispanic | 0.86 (0.35-2.13) | 1.31 (0.52-3.31) | 1.27 (0.50-3.20) |  | 1.48 (0.87-2.51) | 1.39 (0.82-2.37) | 1.33 (0.78-2.27) |
| Other^e^ | 1.57 (0.42-5.95) | 4.98 (1.78-13.93) | 4.80 (1.73-13.28) |  | 1.51 (0.66-3.45) | 0.97 (0.35-2.71) | 0.93 (0.31-2.82) |
| **Non-cancer mortality** | |  |  |  |  |  |  |
| Non-Hispanic White | ref. | ref. | ref. |  | ref. | ref. | ref. |
| Non-Hispanic Black | 1.19 (0.54-2.60) | 1.57 (0.59-4.15) | 1.60 (0.56-4.52) |  | 1.21 (0.56-2.60) | 1.15 (0.51-2.58) | 1.03 (0.45-2.38) |
| Hispanic | 1.69 (0.46-6.19) | 1.73 (0.34-8.74) | 1.93 (0.43-8.75) |  | 0.89 (0.23-3.42) | 0.54 (0.10-2.90) | 0.49 (0.09-2.77) |
| Other^e^ | NA | NA | NA |  | NA | NA | NA |
| Abbreviations: CI = confidence interval; HR = hazard ratio; ref. = referent; MMTP = Medical Monitoring and Treatment Program; NA = not available due to no events in the subgroup; SES = socioeconomic status | | | | | | | |
| ^a^Excluded those with unknown data on MMTP status for 268 (out of 1768) rescue and recovery workers in the World Trade Center Health Registry. | | | | | | | |
| ^b^Model is stratified by age group at diagnosis, but unadjusted for covariates. | | | |  |  |  |  |
| ^c^Model is stratified by age group at diagnosis and adjusted for gender, smoking status, PCL score, stage at diagnosis, grade at diagnosis, amenability, second cancer, and pre-911 physical health conditions. | | | | | | | |
| ^d^Model is stratified by age group at diagnosis and adjusted for gender, smoking status, PCL score, stage at diagnosis, grade at diagnosis, amenability, second cancer, pre-911 physical health conditions, and socioeconomic status. | | | | | | | |
| ^e^Other includes non-Hispanic Asian or Pacific Islander, Native American, and other participant responses. | | | | | | | |
